# Adhered ECMO cannula in COVID-19 related severe acute respiratory failure

**DOI:** 10.1186/s13019-022-02004-4

**Published:** 2022-10-08

**Authors:** Mohammed A. Kamalia, Samuel F. Carlson, Joshua Melamed, Adam Ubert, Peter J. Rossi, Lucian A. Durham

**Affiliations:** 1 Medical College of Wisconsin, Milwaukee, WI USA; 2 Department of Surgery, Division of Cardiothoracic Surgery, Medical College of Wisconsin, HUB for Collaborative Medicine, 5th Floor, 8701 Watertown Plank Rd., Milwaukee, WI 53226 USA; 3 Department of Surgery, Division of Vascular and Endovascular Surgery, Medical College of Wisconsin, Milwaukee, WI USA

**Keywords:** VV ECMO, Crescent, Dual-lumen cannula, Extracorporeal membrane oxygenation, Severe acute respiratory failure, Case report

## Abstract

**Background:**

Crescent cannula adhesion in the setting of COVID-19 respiratory failure requiring extracorporeal membrane oxygenation (ECMO) support is a novel complication. The objective of this case presentation is to highlight this rare complication and to explore potential predisposing factors and our management strategies.

**Case presentation:**

We present the case of a 25 y.o. patient with COVID-19 respiratory failure requiring ECMO support for 16-days in which a 32 Fr crescent cannula became adherent to the SVC and proximal jugular vein. Attempts to remove the cannula at the bedside failed due to immobility of the cannula. Ultrasound of the right neck was unremarkable, so he was taken to the hybrid OR where both TEE and fluoroscopy were unrevealing. An upper sternotomy was performed, and the superior vena cava and proximal jugular vein were dissected revealing a 2 cm segment of the distal SVC and proximal jugular vein that was densely sclerosed and adherent to the cannula. The vessel was opened across the adherent area at the level of the innominate vein and the cannula was then able to be withdrawn. The patient suffered no ill effects and had an unremarkable recovery to discharge.

**Conclusions:**

To date, there have been no reports of crescent cannula adhesion related complications. In patients with COVID-19 respiratory failure requiring ECMO, clinicians should be aware of widespread hypercoagulability and the potential of unprovoked, localized venous sclerosis and cannula adhesion. We report our technique of decannulation in the setting of cannula adhesion and hope that presentation will shed further light on this complication allowing clinicians to optimize patient care.

## Background

Extracorporeal membrane oxygenation (ECMO) support is a valuable treatment modality for terminally ill patients with refractory COVID-19 infection [1]. However, known complications of ECMO cannulation include migration, dislodgement and inadvertent decannulation. Thus, various tissue adhesives have occasionally been utilized to secure the cannula at the insertion site [2]. Additionally, infection with COVID-19 has been shown to cause multisystem derangements, including systemic arterial and venous thromboembolism [3]. The etiology remains unclear, but it is postulated that inflammation secondary to COVID-19 infection leads to a systemic inflammatory response syndrome (SIRS) [4]. The resulting cytokine storm and inappropriate activation of the coagulation cascade leads to increased likelihood of acute thrombosis [5].

We present the case of a 25-year-old patient with COVID-19 respiratory failure requiring ECMO support for 16-days complicated by adhesion of a 32 Fr crescent cannula to the lumen of the superior vena cava (SVC). To date, there have been no reports of crescent cannula adhesion related complications.

## Case presentation

A 25-year-old male presented to an outside emergency department (ED) with a five-day history of cough, shortness of breath, nasal congestion, fatigue and loss of taste and smell. He had a past medical history of asthma and morbid obesity (BMI 50.5 kg/m^2^). The patient had no known connective tissue disease or hypertrophic scarring reactions. He had a 2-year history of vaping but states he quit one month prior to presentation. Sick contacts include a brother and his mother who tested positive for COVID-19 four days prior. He had a positive COVID-19 test three days prior to admission. In the ED, he was hypoxic to 88% on room air and tachycardic at 133 bpm. He improved to 90% saturation on 3 L nasal cannula (NC). Venous blood gas revealed normal venous pH of 7.4 and increased PCO2 of 50. Chest x-ray (CXR) revealed bilateral airspace disease consistent with known COVID-19 infection. He was admitted to the MICU and treated with Remdesivir and high dose steroids. He became febrile to 102.9 F, tachycardic to 134 bpm, tachypneic to 28, and required 15 L O_2_ via high-flow nasal cannula. D-dimer was normal at 0.28. His respiratory status continued to decline over the night of admission, requiring BiPAP with a minute volume of 25 L and respiratory rate of 40. Due to worsening respiratory distress and persistent hypoxia, despite aggressive respiratory support, the patient required intubation.

He was started on ceftriaxone and azithromycin due to SIRS criteria and an elevated procalcitonin. Ferritin and C-reactive protein were mildly elevated, but not unlike what is seen in most COVID patients. Oxygen saturation was 92% on 100% Fi02 via ventilator. Repeat CXR revealed diffuse bilateral interstitial infiltrates. A SARS-COV-2-IGG antibody was non-reactive, and he received a transfusion of convalescent plasma. Oxygen saturation was 53% despite maximal ventilatory support and he was transferred to our institution for ECMO consideration.

**Fig. 1:**
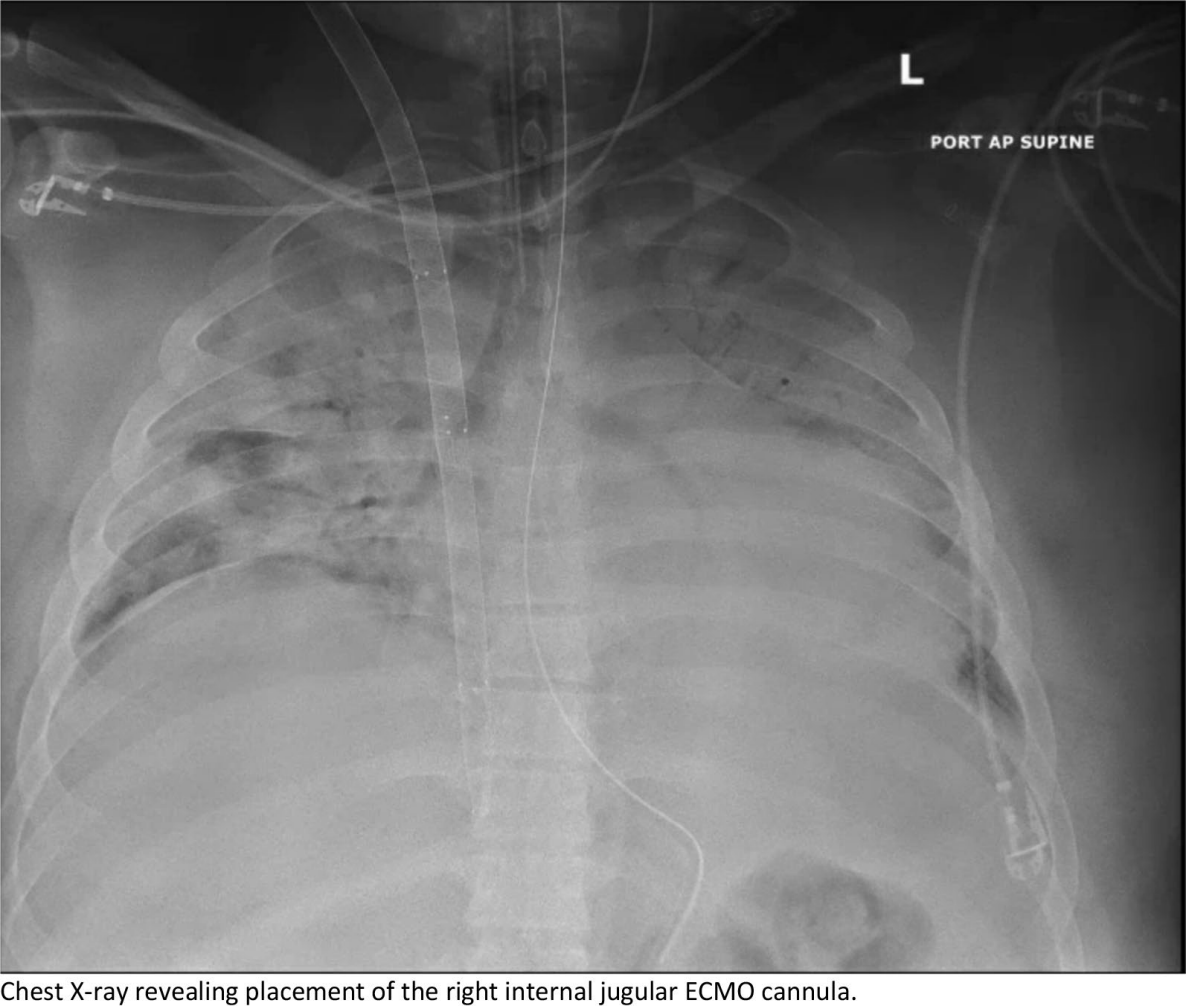
Chest X-ray revealing placement of the right internal jugular ECMO cannula

**Fig. 2:**
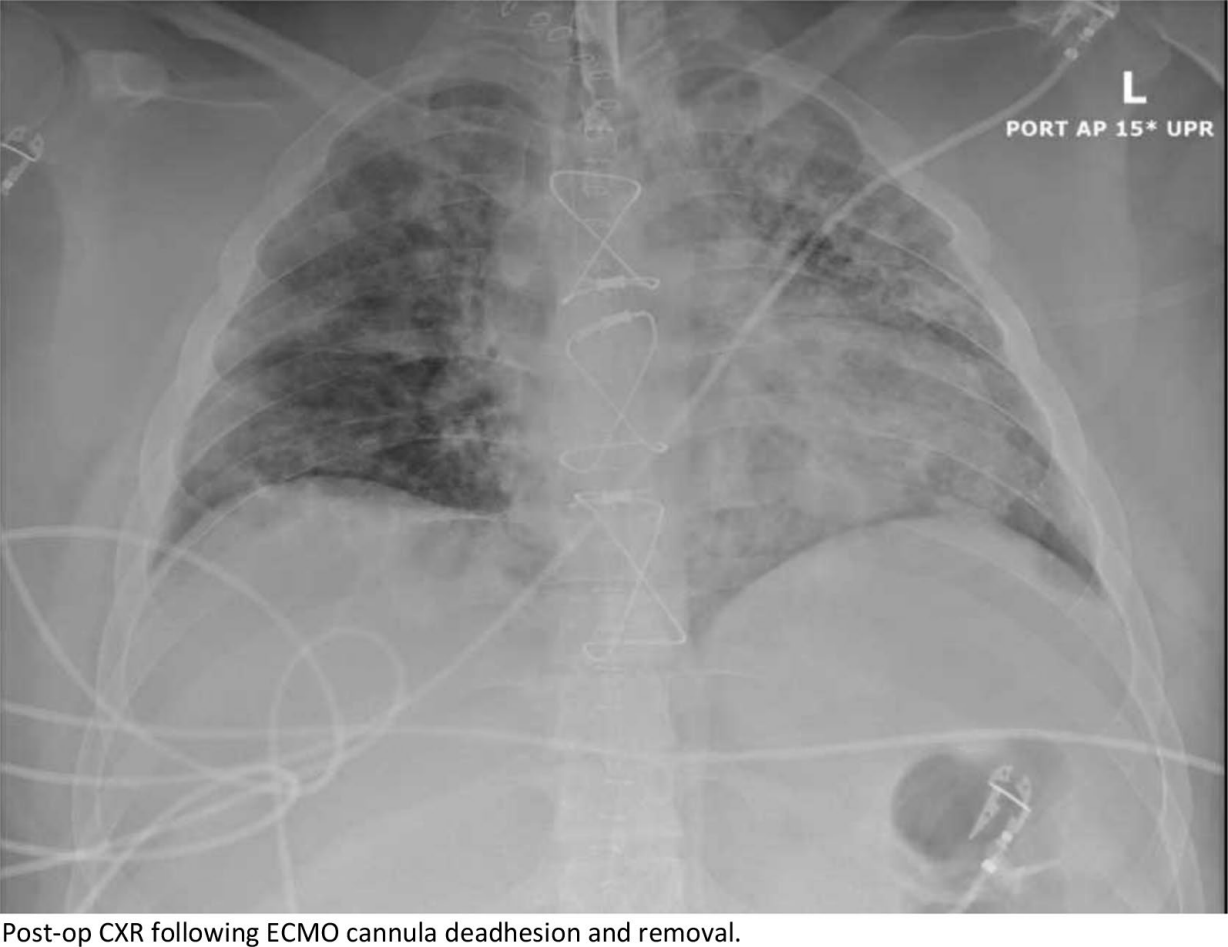
Post-op CXR following ECMO cannula deadhesion and removal

Upon arrival, the patient went from the heliport directly to the hybrid operating room where he underwent unremarkable cannulation with no repositioning required, and initiation of veno-venous ECMO with a 32 Fr Crescent™ double lumen cannula (Medtronic, Inc, Minneapolis, MN, USA) under transesophageal echocardiographic (TEE) and fluoroscopic guidance. There was a single attempt with no repositioning required. No upper body central line was present in juxtaposition with the VV-ECMO cannula. AP CXR is shown in Fig. 1 following ECMO cannulation. The patient was supported on V-V ECMO for 14 days at which time he was able to be weaned gradually from the circuit for bedside decannulation on POD 16. He was maintained on heparin for anticoagulation and chart review of unfractionated heparin lelvels showed that he was never subtherapeutic. Attempts to remove the cannula at the bedside failed due to immobility of the cannula. Ultrasound of the right neck was unremarkable, so he was taken to the hybrid OR where both TEE and fluoroscopy were unrevealing. A longitudinal cutdown from the insertion site proceeding caudally was performed to expose the internal jugular vein. The cannula was freely mobile to the level of the first rib and clavicle and was not obstructing the vein. An upper sternotomy, confined to the manubrium, was performed and the superior vena cava and proximal jugular vein were dissected revealing a 2 cm segment of the distal SVC and proximal jugular vein that was densely sclerosed and adherent to the cannula. Proximal and distal control of the vasculature was established, and the vessel was opened across the adherent area at the level of the innominate vein. The cannula was then able to be withdrawn without difficulty. There was no evidence of thrombosis, scarring or local inflammation at the site of cannula adhesion. The distal SVC was repaired to the level of the innominate vein entry to the SVC to ensure good drainage, however, due to the severe sclerosis and damage to the jugular above the innominate, the vein was ligated proximally and distally. Post-op CXR is shown in Fig. 2. The patient suffered no ill effects and had an unremarkable recovery to discharge. Clinic follow up has also demonstrated no sequalae or serious adverse events. The Crescent catheter was examined by pathology at our institution and then returned to the company, neither found any defects. At our institution, we use Avalon, Crescent and ProTek Duo cannulas and have never experienced adhesions with any of the cannulas.

## Discussion

COVID-19 infection can result in widespread hypercoagulability including acute arterial and venous thromboembolism [5]. Additionally, presence of foreign body within the vasculature may initiate an inflammatory response serving as a lead point for a novel and unreported complication of ECMO cannulation, such as unprovoked, localized venous sclerosis and cannula adhesion [6]. To date, there have been no reports of crescent cannula adhesion related complications.

The use of a percutaneous double lumen cannula for veno-venous ECMO (V-V ECMO) offers several advantages over peripheral ECMO, including ease of cannulation, patient comfort, earlier mobilization and physiotherapy [7]. Fleet et al. analyzed 54 patients with severe acute respiratory failure requiring V-V ECMO support using the crescent cannula. All insertions were via right internal jugular vein (IJV) and most utilized a 32 Fr cannula. They found it to be both safe and effective with no life-threatening injuries and a low number of minor cannula-related complications such as cannula repositioning and requiring placement of an additional drainage cannula [8].

It is very uncommon for a large-bore central venous cannula to be inserted easily, and then be difficult or impossible to remove. A systematic review in 2015 evaluated 25 studies including 14,107 patients that suffered venous thrombosis in the setting of an indwelling central venous catheter, finding that increasing BMI, advancing age, chemotherapy administration, and malignancy were associated with thrombosis [9], which could theoretically make cannula removal more difficult. However, no studies in the English language literature provide further insight into the complication in our patient.

The patient in this case did not demonstrate venous thrombosis, but instead had a fibrotic segment of the right innominate vein. This may have been pre-existing or may have developed as an inflammatory reaction to the cannula itself. While chronic SVC thrombosis has been reported frequently in the setting of indwelling catheters, including a previous report of catheters becoming “stuck” [10], this has not been reported in the acute setting. In this patient, adhesion is likely a result of the inflammation from active COVID-19 infection and disruption of the coagulation system. Factors such as catheter insertion site, length of catheter, gender, previous catheter presence, and catheter composition are all possible contributing factors to the development of catheter adherence [11, 12].

It is well known that COVID-19 infection is associated with vascular thrombosis [13]. Hyperinflammation associated with infection has been hypothesized to contribute to contribute to many vascular complications, namely thrombosis. Derangement of blood flow through the venous and arterial vasa vasorum is one such theory. While data is sparse, Pasquinelli et al. showed, through histological analysis, that adventitial microcirculation is a major target of SARS-COV-2 mediated vascular inflammation. In their case they found that endothelial inflammation was most prevalent in the perivascular microcirculation as endothelial cells are primary targets of the SARS-Cov-2 virus [14]. It is impossible to know for fact, but in our patient, there may have been a component of localized microcirculatory thrombosis and subsequent adventitial fibrosis that resulted in our cannula becoming adhered to the luminal wall.

When dealing with an adhered catheter, complications during removal can include vascular damage, rupture, and retention of catheter fragments [11]. Methods of removal include gentle traction, cutaneous cut-down, and rotation of the catheter. One of the best-known techniques is endoluminal dilation involving positioning a balloon within a catheter, expanding the lumen, and stretching the catheter wall. This disrupts the fibrin sheath and adhesions between the catheter and the vein. In our case we performed an upper sternotomy as the catheter was tightly adhered to the SVC, where positioning of and subsequent dilation using a balloon would likely have resulted in SVC perforation.

## Conclusion

A novel and unreported complication in a patient with COVID related respiratory failure requiring ECMO support for 16-days is cannula adhesion. We report a case of a 32Fr crescent cannula adhered to the lumen of the SVC during decannulation requiring an upper sternotomy for surgical removal. The patient suffered no ill effects and demonstrated no sequalae or serious adverse events on discharge and clinic follow-up. Given the extremely rare occurrence of a stuck ECMO cannula, it is challenging to develop evidence-based management strategies, however cardiac surgeons and critical care physicians should be aware of this complication and its subsequent endovascular and open surgical management. Presentation of this case will open discussion on management of similar complications and help optimize treatment strategies or even expedite care for patient with similar issues in the future.

## Data Availability

As this paper is a case report, all data generated or analyzed are included in this article.

## Abbreviations

CXR: Chest X-ray
CT: Com
ECMO: Extracorporeal membrane oxygenation
V-V ECMO: Veno-venous extracorporeal membrane oxygenation
IJV: Internal jugular vein
SVC: Superior vena cava
NC: Nasal cannula
TEE: Transesophageal echocardiogram
